# Immunophenotypic Analysis of T Lymphocytes and Cytokine Production in Elderly Practicing Physical Activities and Its Relationship with Quality of Life and Depression

**DOI:** 10.1155/2022/7985596

**Published:** 2022-09-22

**Authors:** Tamires Marielem de Carvalho-Costa, José Rodrigues do Carmo Neto, Anna Glória Fonseca Teodoro, Flávia Zero Soares, Luiza Pimenta Rochael, Thaís Farnesi Soares de Assunção, Beatriz Coutinho de Souza, Beatriz Sodré Matos, Paula Degani Ferreira dos Santos, Djalma Alexandre Alves Silva, Juliana Reis Machado, Paulo Roberto da Silva, Monique Gomes Salles Tiburcio-Costa, Carlo José Freire de Oliveira, Virmondes Rodrigues Júnior, Denise Bertulucci Rocha Rodrigues, Marcos Vinícius da Silva

**Affiliations:** ^1^Department of Microbiology, Immunology and Parasitology, Institute of Biological and Natural Sciences, Federal University of Triângulo Mineiro, Uberaba, MG, Brazil; ^2^Department of Bioscience and Technology, Institute of Tropical Pathology and Public Health, Federal University of Goias, Goiania, GO, Brazil; ^3^Department of Pathology, Genetics and Evolution, Institute of Biological and Natural Sciences, Federal University of Triângulo Mineiro, Uberaba, MG, Brazil; ^4^Cefores, Federal University of Triângulo Mineiro, Uberaba, MG, Brazil; ^5^Federal University of Lavras, Lavras, MG, Brazil; ^6^Laboratory of Biopathology and Molecular Biology, University of Uberaba, Uberaba, MG, Brazil

## Abstract

Aging is a complex process often associated with a chronic inflammatory profile that alters several biological functions, including the immune system and cognitive and physical capacity. The practice of physical activity is increasingly gaining popularity as a method of preventing infections, depression, and other disorders that affect the quality of life of the elderly. Thus, this work analyzes the profile of cytokines and molecular markers expressed in immune cells of elderly people who practice physical activities or not, evaluating their impacts on the immune system and quality of life. For this, 48 individuals were recruited, and peripheral blood samples were collected for hemogram analysis, cytokine determination, and immunophenotyping. Elderly people were separated into two groups: practitioners with low-intensity physical activity and non-practitioners. Quality of life was assessed using the Whoqol-Old instrument, and depression was assessed using the Beck II Depression Inventory. When comparing the scores of the Whoqol-Old and Beck questionnaires, we observed a significant negative correlation between these two factors. The perception of a higher quality of life was present in the elderly who exercised and was related to greater autonomy and sensory abilities, whereas the presence of depression was lower. In the hemogram, we observed higher basophil and segmented counts in the sedentary elderly, whereas lymphocytes and monocytes had lower counts. Elderly practitioners of physical activities had higher levels of IFN-*γ*, IL-4, and IL-10; increased expression of CD69, PD1, and TIM-3 in CD4+ T lymphocytes and increased CD14+CD80+ and CD14+CD86+ monocytes. Elderly people with an increased perception of quality of life had higher levels of IFN-*γ*, higher expression of CD14+CD80+CD86+, and decreased levels of TRAIL. An increase in TRAIL was observed in individuals with depression, in addition to an increased expression of CD14+CD86+. These results show a clear correlation between the quality of life, level of depression, physical activity, and immune system function. Although some cytokines with a typical proinflammatory profile (IFN-*γ*) were observed, the results point to a protective state with benefits reflected in the general well-being of the elderly who exercise.

## 1. Introduction

In general, aging is associated with complex changes in the body, including the immune system. These changes result from interactions with the external environment resulting in exposure to pathogenic microorganisms, poor quality of life, and genetic alterations [1]. In addition, early disability induced by loss of function and muscle mass in the elderly may be associated with chronic inflammatory processes commonly present during aging, known as the subclinical inflammatory state [2].

Another problem that affects many elderly people and contributes to reduced quality of life and disability is depression [3], a multifactorial disease that has grown worldwide. The elderly have many characteristics that favor their development [4], including the presence of chronic diseases [5] that lead them to lose their routine and independence. In addition, the symptoms of depression are strongly associated with the risk of morbidity during aging.

Drug treatments provide temporary relief and cannot be applied for prolonged periods owing to their side effects [6]. Thus, regular physical activity has been an explored alternative, bringing benefits related to the physiological improvement of the systems and the maintenance of the physical condition, preventing situations that generate disability in the elderly [6, 7]. The practice of physical activity can also help in the alternative treatment for depression [8], reducing symptoms, and improving the quality of life of the elderly [9].

In this context, this study analyzed the profile of cytokines and molecular markers expressed in the immune cells of elderly people who practice physical activities.

## 2. Materials and Methods

### 2.1. Participants

Forty-eight individuals aged 60 years or older (median 63.5, Min/Max 60/86), including 27 men and 21 women, were recruited during care at the Elderly Care Unit (UAI) in Uberaba, state of Minas Gerais, Brazil. UAI is a free municipal public space focused on the psychosocial well-being of the elderly, where they can perform different recreational physical and mental activities. Two groups were formed: elderly practitioners of physical and/or mental health (*n* = 15) and elderly individuals considered sedentary (*n* = 33). The type, intensity, and duration of physical and mental activity were assessed using the International Physical Activity Questionnaire-Short Form and patient self-reports. All elderly practitioners of regular physical and/or mental health performed low-to moderate-intensity activities, with an average duration of 55 minutes per day, three times a week. The physical or mental activities performed were hydrogymnastics (*n* = 9), gymnastics (*n* = 5), dancing (*n* = 4), weight training (*n* = 3), swimming (*n* = 3), snookering (*n* = 3), volleyball (*n* = 2), basketball (*n* = 1), capoeira (*n* = 1), and board games (*n* = 1). The comorbidities included systemic arterial hypertension (*n* = 8), dyslipidemia (*n* = 4), diabetes mellitus (*n* = 2), Chagas disease (*n* = 2), hypothyroidism (*n* = 2), arthrosis (*n* = 1), stroke (*n* = 1), back pain (*n* = 1), glaucoma (*n* = 1), insomnia (*n* = 1), and labyrinthitis (*n* = 1). This study was approved by the ethics committee for research with human beings of the Federal University of Triângulo Mineiro, and all participants signed the free consent form after clarification. This study was approved by the Research Ethics Committee of the Federal University of Triângulo Mineiro (CEP/UFTM/2390/2012).

### 2.2. Assessment of Individual Perception of Quality of Life and Assessment of the Depression Scale

The Whoqol-Old instrument was used to assess individual perceptions of the quality of life [10]. This questionnaire has 24 questions, grouped into six facets: sensory functioning; autonomy; past, present, and future activities; social participation; death and dying; and intimacy. The answers followed a Likert scale (from 1 to 5), and, according to the scores assigned to each question, a score ranging from 4 to 20 was obtained for each facet, which was later corrected to result in percentages. Higher scores represent a higher quality of life. The scores of these six facets combined provided an overall score separating the quality of life of the elderly into three categories: good (80–100), fair (60–80), and need for improvement (0–60).

The assessment of the depression scale was performed using the Beck II Depression Inventory (BDI) [11, 12]. Through 21 items, this questionnaire sought to measure the severity of depressive symptoms in two scenarios common to the elderly: cognitive-affective and physical-somatic scenarios. The assessment of the intensity of depressive symptoms is reflected in the score (BDI score) obtained by each of the elderly and classified as no depression (0–13), mild depression (14–19), moderate depression (20–28), and severe depression (29–63).

### 2.3. Blood Count and Determination of Serum Cytokine Levels

Two peripheral blood samples were collected for blood count assessment and serum cytokine quantification. For this, a sample was collected in a tube containing EDTA (for the blood count) and another in a tube without anticoagulant (for cytokine quantification).

The blood count was performed using an automated method and the serum levels of cytokines IL-1*β*, IL-4, IL-6, IL-10, IFN-*γ*, TNF-*α*, TGF-*β*, and TRAIL were quantified by ELISA (BD Pharmingen-USA). The concentration of cytokines was determined by linear regression with the absorbance obtained from the recombinant cytokines and expressed in pg/mL, always respecting the detection limit of each assay.

### 2.4. Evaluation of the Expression of Surface Molecules of CD4^+^ and T CD8^+^ Lymphocytes by Flow Cytometry

For immunophenotyping, a third sample was collected in a tube containing heparin. The circulating lymphocytes of the patients were characterized by their subpopulation between auxiliary and cytolytic (CD4+ or CD8+, respectively), concerning the expression of the activation marker (CD69), in addition to coinhibitory molecules (TIM-3 and PD-1). The monocytes were evaluated for the expression of CD14, CD80, and CD86.

Briefly, whole blood samples collected in heparinized tubes were blocked with phosphate-buffered saline containing 10% inactivated AB+ human serum. Specific monoclonal antibodies directed to the surface molecules of lymphocytes were then added (anti-CD4, anti-CD8, anti-CD69, anti-TIM-3, anti-PD-1, anti-CD14, anti-CD80, and anti-CD86) conjugated to different fluorophores (FITC, PE, and PE-Cy5) and combined according to the convenience of the assay. When reading the assay, lysis of red blood cells was performed, and the cells were acquired using a FACS-CALIBUR cytometer (Becton & Dickinson, USA). The analysis of the acquired cells was performed using the Cell Quest program (Becton & Dickinson, USA), from the individualization of the leukocyte population through gates established according to the size (FSC) and granularity (SSC) standards and analyzed using FlowJo 10 software (Becton, Dickinson & Company).

### 2.5. Statistical Analysis

The database was created using the EXCEL 2016 program, and statistical analyses were performed using GRAPHPAD PRISM 8.0.1 (GRAPHPAD SOFTWARE, USA). The verification of the normal distribution of quantitative variables was evaluated using the D'Agostino and Pearson tests. For comparison between the two groups, the unpaired *t*-test was used for data with a normal distribution and the Mann–Whitney test for data with a nonnormal distribution. Correlations were determined using Spearman's test with a 95% confidence interval. The results were considered statistically significant at *p* < 0.05.

## 3. Results

### 3.1. Assessment of Individual Perception of Quality of Life and Depression Scale

**Figure 1:**
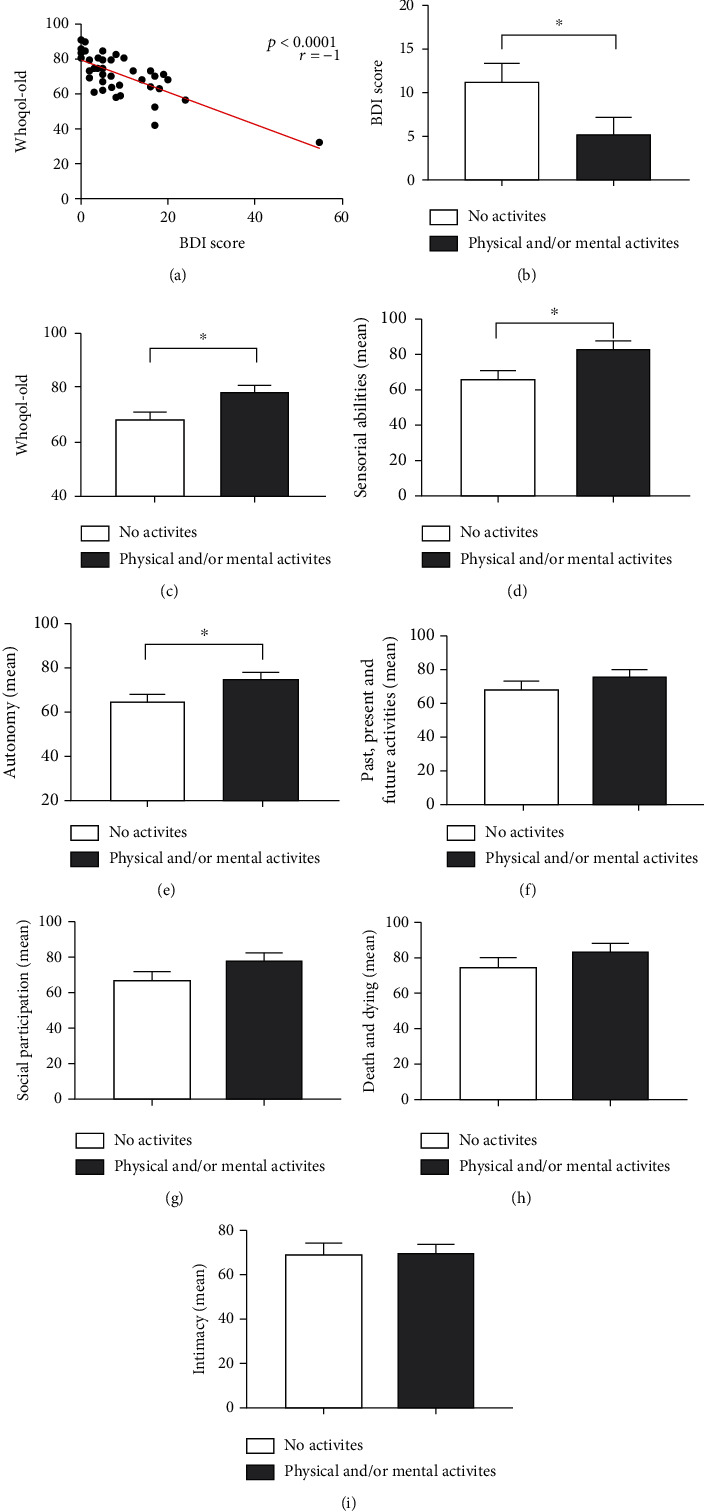
Assessment of individual perception of quality of life, depression scale, and impact of physical and/or mental activity in the elderly. (a) Correlation between the values obtained in the Whoqol-Old questionnaire and the Beck Depression Inventory II. (b) Impact of physical and/or mental activity on individual perception of quality of life (Whoqol-Old). (c) Impact of physical and/or mental activity on assessing the depression scale (BDI score). (D-I) Impact of physical and/or mental activity on the following assessment facets of individual perception of quality of life: sensory functioning; autonomy; past, present, and future activities; social participation; death and dying; and intimacy, respectively. Differences are considered statistically significant when *p* < 0.05.

Of the total number of elderly people evaluated, 34 (70.8%) answered questionnaires to assess their quality of life and depression. According to the Whoqol-Old questionnaire, 14 individuals had a quality of life classified as “good,” 18 as “regular,” and two as “need to improve.” Of the 34 elderly people who responded to the Beck Depression Inventory, 23 had no depression, 10 had mild/moderate depression, and one had severe depression. When comparing the scores of the quality of life (Whoqol-Old) and depression (Beck) questionnaires, it was possible to report a significant, strong, and negative correlation (*p* < 0.0001 and *r* = −1) (Figure 1(a)). Thus, the better the quality of life, the lower the signs of depression.

When comparing the practice of physical activity with the answers obtained in the questionnaires, it was observed that elderly practitioners of physical activity had lower scores on the Beck Depression Inventory (*p* = 0.0280) (Figure 1(b)) and higher values on the Whoqol-Old (*p* = 0.0276) (Figure 1(c)). Interestingly, it was possible to observe that this same group, the physically active elderly, demonstrated greater sensory (*p* = 0.0291) (Figure 1(d)) and autonomy (*p* = 0.0472) skills (Figure 1(e)). Regarding the other facets of the Whoqol-Old test, such as past, present, and future activities (*p* = 0.2623) (Figure 1(f)); social participation (*p* = 0.0779) (Figure 1(g)); death and dying (*p* = 0.3346) (Figure 1(h)); and intimacy (*p* = 0.7289) (Figure 1(i)), there were no statistical differences between the groups.

### 3.2. Hematological Parameters and Determination of Serum Cytokine Levels

**Figure 2:**
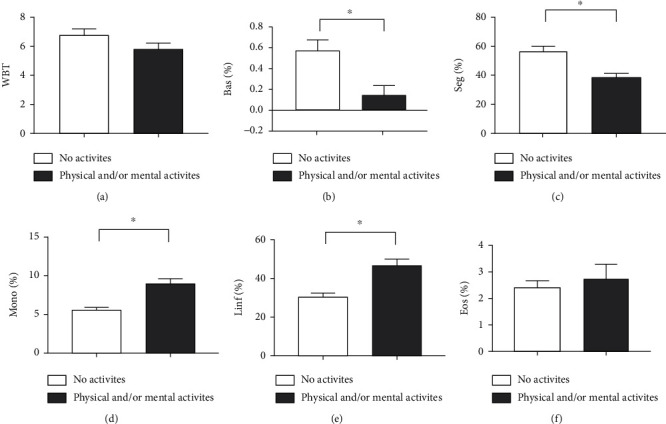
Impact of physical and/or mental activity on hematological parameters in the elderly. (a) total leukocytes (WBT), (b) basophils (bas), (c) segmented (sec), (d) monocytes (mono), (e) lymphocytes (lymph), and (f) eosinophils (eos). Differences are considered statistically significant when *p* < 0.05.

After analyzing the impact of physical activity on the parameters of individual perception of quality of life and on the BDI, the next step was to assess the effects on hematological parameters (Figure 2). Regarding total leukocyte count, there was no statistically significant difference between the groups (*p* = 0.2344) (Figure 2(a)). Interestingly, when the relative percentage of the main circulating leukocyte subpopulations was evaluated, it was observed that physical and/or mental activity induced an increase in basophils (*p* = 0.0005) (Figure 2(b)) and segmented (*p* < 0.0001) (Figure 2(c)). Furthermore, in the same group, monocytes (*p* = 0.0001) (Figure 2(d)) and lymphocytes (*p* < 0.0001) (Figure 2(e)) had lower counts than the group that did not practice physical activity. Finally, eosinophil counts were not significantly different between the groups (*p* = 0.5293) (Figure 2(f)).

**Figure 3:**
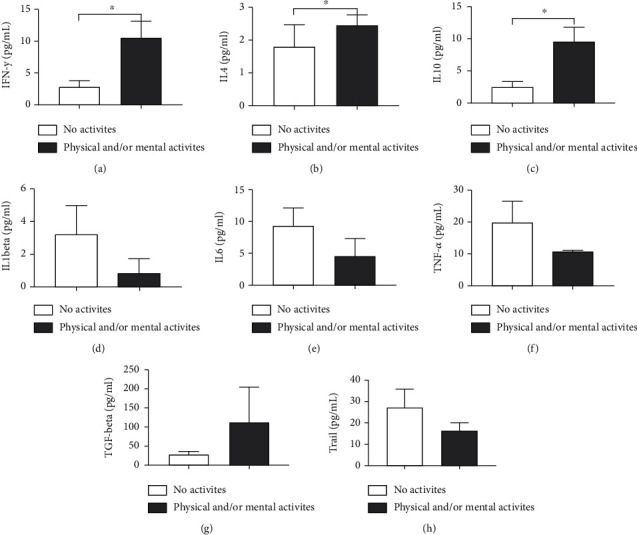
Impact of physical activity on the serum cytokine profile of the elderly. (a) IFN-*γ*, (B) IL-4, (c) IL-10, (d) IL-1*β*, (E) IL-6, (f) TNF-*α*, (g) TGF-*β*, and (h) TRAIL. Differences are considered statistically significant when *p* < 0.05.

Regarding serum cytokines (Figure 3), elderly people who exercised had higher levels of IFN-*γ*(*p* = 0.0002) (Figure 3(a)), IL-4 (*p* = 0.0027) (Figures 3(a) and 3(b)), and IL-10 (*p* = 0.0007) (Figure 3(c)) compared to sedentary individuals. No statistical differences were observed between the groups for IL-1*β* (*p* = 0.5546) (Figure 3(d)), IL-6 (*p* = 0.8882) (Figure 3(e)), TNF-*α* (*p* = 0.5430) (Figure 3(f)), TGF-*β*(*p* = 0.9272) (Figure 3(g)), and TRAIL (*p* = 0.3851) (Figure 3(h)).

**Figure 4:**
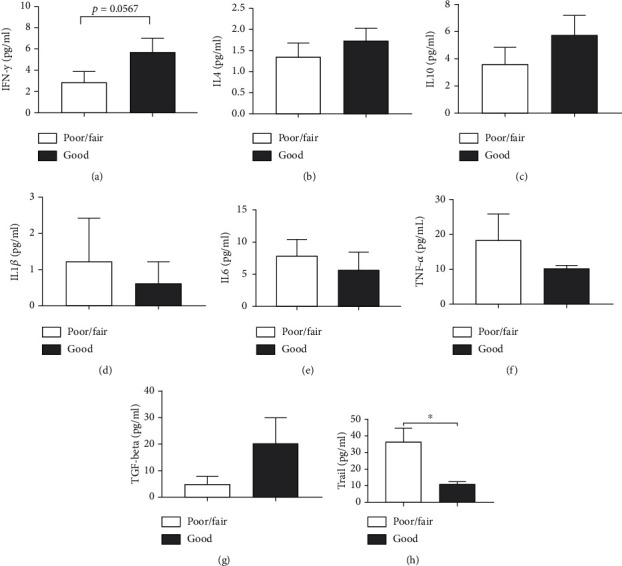
Impact of perceived quality of life on the serum cytokine profile of the elderly. (a) IFN-*γ*, (b) IL-4, (c) IL-10, (d) IL-1*β*, (e) IL-6, (f) TNF-*α*, (g) TGF-*β* and (h) TRAIL. Differences are considered statistically significant when *p* < 0.05.

Elderly people with an increased perception of quality of life (Figure 4) showed a tendency to have increased IFN-*γ* (*p* = 0.0567) (Figure 3(a)) and lower serum levels of TRAIL compared to the poor/fair group (*p* = 0.0011) (Figure 4(h)). For the remaining cytokines evaluated, no statistically significant differences were observed: IL-4 (*p* = 0.4078) (Figure 3(b)), IL-10 (*p* = 0.1511) (Figure 3(c)), IL-1*β* (*p* = 0.4099) (Figure 4(d)), IL-6 (*p* = 0.1090) (Figure 4(e)), TNF-*α* (*p* = 0.6212) (Figure 4(f)), and TGF-*β* (*p* = 0.4562) (Figure 4(g)) (Figure 4(h)).

**Figure 5:**
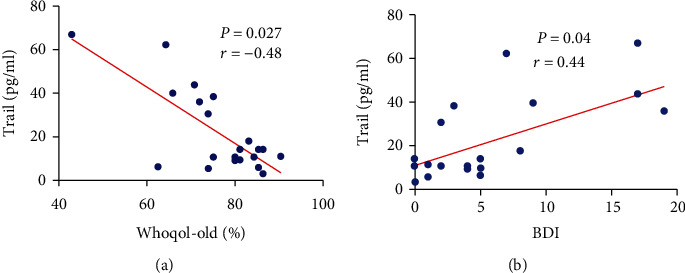
Correlation of TRAIL with (a) individual perception of quality of life (Whoqol-Old score) and with (b) depression scale (BDI). A Spearman correlation test was performed. Differences are considered statistically significant when *p* < 0.05.

Elderly people with a worse quality of life showed an inversely proportional correlation with TRAIL rates (*p* = 0.027 and *r* = −0.48) (Figure 5(a)). Higher values for TRAIL were also those that presented with more depression according to the BDI (*p* = 0.04 and *r* = 0.44) (Figure 5(b)).

### 3.3. Evaluation of the Expression of Surface Molecules of CD4+ and T CD8+ Lymphocytes by Flow Cytometry

**Figure 6:**
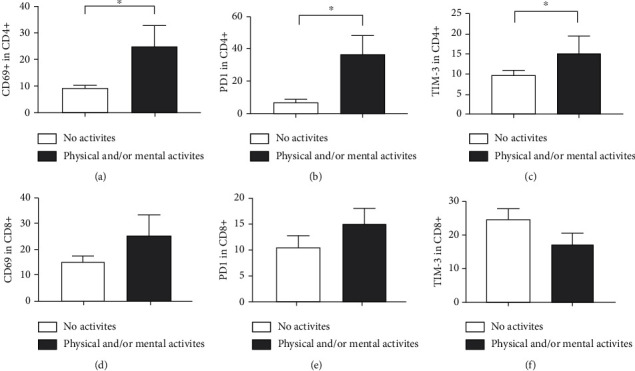
Impact of physical activity on the expression profile of surface markers in CD4+ and CD8+ T lymphocytes obtained from the blood of elderly people. Expressions of (a) CD69, (b) PD1, and (c) TIM-3 on CD4+ T lymphocytes. Expressions of (d) CD69, (e) PD1, and (f) TIM-3 on CD8+ T lymphocytes. Differences are considered statistically significant when *p* < 0.05.

When evaluating markers on the surface of T lymphocytes (Figure 6), it was observed that individuals who practiced physical activity showed a greater number of CD69+ (*p* = 0.0062) (Figure 6(a)) and PD1 + (Figure 6(b)) cells (*p* = 0.0008) CD4+ cells but not CD8+ cells (*p* = 0.3435 and *p* = 0.2672, respectively) (Figures 6(d) and 6(e)). Furthermore, there was no statistical difference in TIM-3 expression in both CD4+ (*p* = 0.1042) (Figure 6(c)) and CD8+ (*p* = 0.2167) (Figure 6(f)) lymphocytes between the two groups.

**Figure 7:**
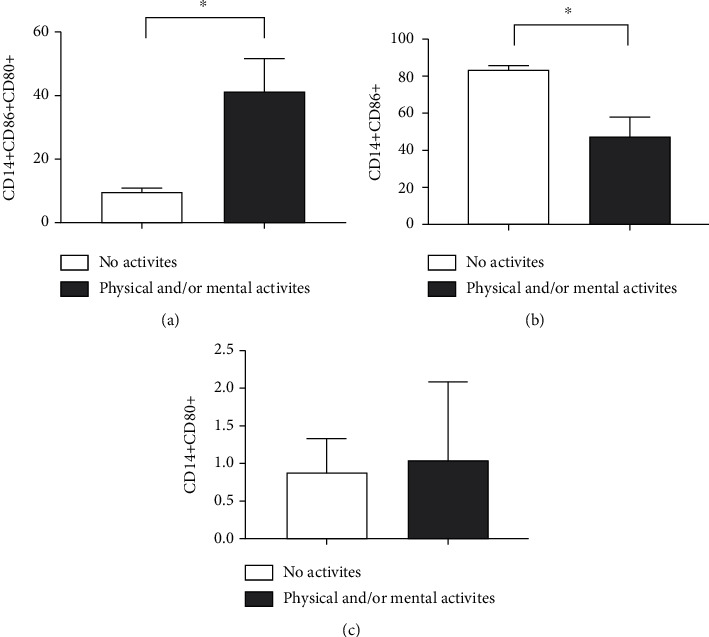
Impact of physical activity on the expression profile of surface markers on CD14+ cells obtained from the blood of the elderly. Expressions of (a) CD14+CD86+CD80+, (b) CD14+CD86+, and (c) CD14+CD80+ on CD14+ cells. Differences are considered statistically significant when *p* < 0.05.

CD14+ cells doubly positive for CD80 and CD86 were found in significantly higher amounts in physically active practitioners (*p* < 0.0001) (Figure 7(a)). When analyzing these two markers separately, we observed that CD86+ showed reduced expression in these individuals (*p* = 0.0002) (Figure 7(b)), whereas CD80+ showed no significant difference between the groups (*p* = 0.1986) (Figure 7(c)).

**Figure 8:**
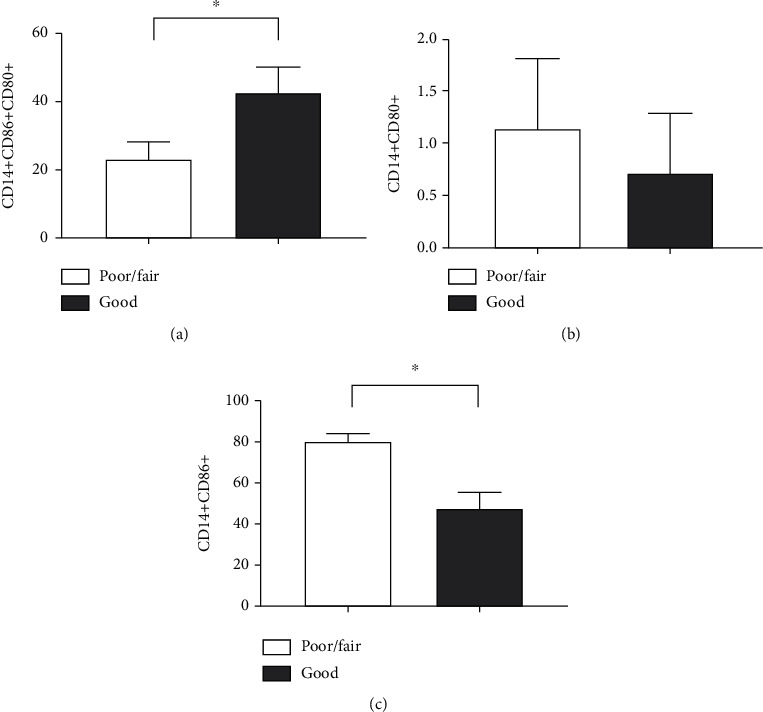
Impact of quality of life perception on the expression profile of surface markers in CD14+ cells obtained from the blood of the elderly. Expression of (a) CD14+CD86+CD80+, (b) CD14+CD80+, and (c) CD14+CD86+. Differences are considered statistically significant when *p* < 0.05.

Regarding the perception of quality of life (Figure 8), elderly people who were classified as having a good quality of life had a higher number of CD14+CD80+CD86+ cells (*p* = 0.0128) (Figure 8(a)) than those classified as poor/fair. There was no significant difference between the groups in the CD14+CD80+ subpopulations (*p* = 0.3638) (Figure 8(b)). However, sedentary elderly individuals showed a higher level of CD14+CD86+ cells (*p* = 0.0007) (Figure 8(c)).

**Figure 9:**
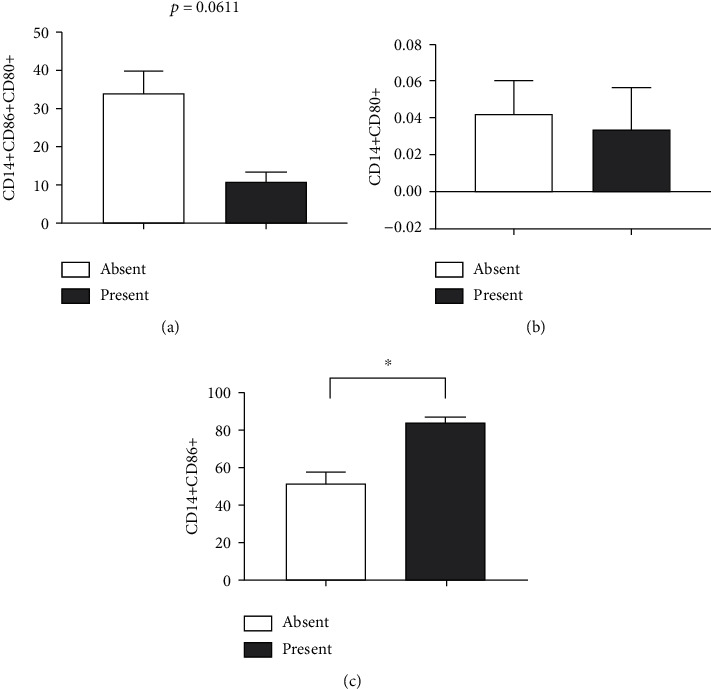
Impact of depression on the expression profile of surface markers on CD14+ cells obtained from the blood of elderly people. Expressions of (a) CD14 +CD86 + CD80+, (b) CD14+CD80+, and (c) CD14+CD86+. Differences are considered statistically significant when *p* < 0.05.

For the elderly with some level of depression (Figure 9), triple-positive cells for CD14+CD80+CD86+ showed a decreasing trend (*p* = 0.0611) (Figure 9(a)). Only those marked by CD14+CD86+ were increased in this group (*p* = 0.0061) (Figure 9(c)). In addition, there was no change in those marked CD14+CD80+ (*p* = 0.8470) (Figure 9(b)) between the groups.

## 4. Discussion

The inflammatory pathway is a potential target for reducing disability resulting from a persistent subclinical inflammatory state, commonly observed in the elderly [13]. Studies have shown that in this population, proinflammatory cytokines are naturally higher compared to the levels seen in middle-aged or young people [14]. Thus, one of the ways to improve this situation is the practice of regular physical activity because there is a good relationship between regular exercise and the serum reduction of proinflammatory cytokines [15]. However, little is known regarding the mechanisms involved in this process.

Among the hypotheses raised, (1) it has been reported that during physical training, the muscles undergo biochemical changes that result in the reduction of the local gene expression of proinflammatory cytokines, such as IL-6, TNF-*α*, and IL- 1*β* [16, 17]. (2) The increase in muscle strength has also been related to lower serum levels of cytokines such as IL-6 and TNF-*α* [18]. (3) Along with the two hypotheses highlighted above, physical activity may induce lower production of proinflammatory cytokines by immune cells [19]. However, immunomodulation seems to depend on the type of exercise, intensity, frequency, and the individual's health condition because there are reports in which physical activity does not influence the serum levels of cytokines [20]. In our study, IL-6, TNF-*α*, and IL-1*β* levels did not differ between the groups, which may be related to the difference in the type of exercise, intensity, and frequency performed among the participants and the time of blood collection for cytokine measurement (before, during, or after physical activity). Despite this, we observed an increase in IL-4 and IL-10 cytokines in the elderly who exercised, suggesting that exercise has a protective anti-inflammatory role [21] and possibly an immune balance (IFN-*γ* and IL-4/IL-10).

The increase in cells, such as lymphocytes and monocytes, in the blood count of the elderly that exercise corroborates the increased production of the cytokine profile. IFN-*γ*, a proinflammatory cytokine, is mainly synthesized by T lymphocytes. This cytokine generally activates macrophages and other immune cells to produce chemokines that recruit more cells to the inflamed site [22]. Although this cytokine is considered a stimulator of inflammation, proinflammatory cytokines can also play immunoregulatory roles, depending on the moment, the amount, and the disease model evaluated [23]. A fact that occurs, for example, during acute exercise, where there is an increase in the production of IL-6 to regulate the output of TNF-*α* and IL-1*β* and increase the production of IL-10, which helps in cellular homeostasis [21, 24]. In addition, it favors lipolysis and glucose homeostasis, consequently stimulating lean mass gain instead of fat [25]. Studies assessing the presence of exercise throughout life show the various benefits involved in the balance of pro- and anti-inflammatory cytokines resulting from muscle activity [26], which may also be reflected in the quality of life of people who practice physical activity [27].

TIM-3+, a surface marker of immunosenescence, especially T lymphocytes, was found to have a higher expression of CD4+ lymphocytes in physically active elderly individuals. This increase may be related to the good results obtained for autonomy and sensory parameters because studies have shown that high levels of CD4+ and CD8+ TIM3+ T cells are inversely associated with the evolution of rheumatoid arthritis [28, 29]. This is a disease that affects many elderly individuals and is responsible for motor impairment in these individuals [30]. Low levels of TNF-*α* may also have contributed to this result [23].

Another interesting point was that the sedentary elderly had a worse perception of quality of life and developed some depression. This may be related to the fact that in elderly people, the subclinical chronic inflammatory state, common during the aging process, is associated with changes in the enzymatic pathways of monoamine metabolism. These molecules directly interfere with the production of serotonin, a substance that gives feelings of well-being [31, 32]. Tryptophan, for example, is a monoamine whose degradation is increased during inflammatory processes caused by stimulation by IFN-*γ*. Thus, an association between inflammatory processes and depressive symptoms is common [31]. In contrast, our findings point to an increase in IFN-*γ* in the elderly who exercise, a fact also found in the work of [33]. In this case, the authors argue that the types of exercise practiced by the evaluated individuals are responsible for these divergent levels. The good mood, perception of increased quality of life, and absence of depressive symptoms observed here are probably caused by the increase in neurotransmitter synthesis or other monoamines that may not bind to tryptophan under the conditions studied.

In our study, TRAIL was inversely correlated with quality of life and directly correlated with depression. Although the role of this cytokine in inflammation is not yet clearly defined [34, 35], studies have reported that this molecule can induce proinflammatory cytokines, such as IL-1*β*, IL-6, and TNF-*α*, in macrophages in vitro [36]. Furthermore, serum TRAIL levels are associated with TNF-*α* and decreased lung function in patients with chronic obstructive pulmonary disease [37]. Both studies support a proinflammatory function. Thus, the negative correlation between TRAIL and quality of life may be associated with the maintenance of an inflammatory profile in sedentary elderly individuals because an increase in anti/regulatory cytokines was not observed in this group. However, an increase in inflammatory cells was observed.

There was no relationship between this cytokine and physical activity. Although the impact of exercise on TRAIL levels is not known, there was no change in this molecule in individuals with atherosclerosis undergoing physical training [38]. More studies related to this topic are needed to elucidate the role of TRAIL better.

Physical activity increased CD69+CD4+ cells and CD80+ and CD86+ cells in our study. In addition, physically active individuals with a good perception of quality of life also showed higher levels of CD14+, CD80+, and CD86+ cells. These markers represent phenotypes of activated T cells [39] and macrophages [40, 41]. It has already been demonstrated that different types of physical exercise induce proliferation, increase, and activate lymphocytes and macrophages [1], including the same molecules evaluated in our work. In addition to activating these cells, physical activity increases in other cell types, such as monocytes, neutrophils, and natural killer cells, especially during and after acute exercise.

Although the study encompasses a small number of participants, which is a limitation in the study that needs to be expanded to cover an individual's larger number, this study showed a clear correlation between the quality of life, level of depression, physical activity, and immune system. Although some cytokines with a typical proinflammatory profile (IFN-*γ*) were observed, the results point to a protective state with evident benefits reflected in the general well-being of the elderly who exercise.

## Data Availability

The original contributions of this study are included in this article. Further inquiries can be directed to the corresponding author.
